# Books Review

**Published:** 1870-04

**Authors:** 


### Books Review.

*A Practical Treatise on the Diseases of Children.* BY J. FORSYTH MEIGS, M. D., and WILLIAM PEPPER, M. D., Philadelphia. LINDSAY & BLAKISTON.

This is the fourth edition of Meigs on the diseases of children, greatly enlarged and improved by chapters upon a large number of new subjects, and also by a very copious index, which facilitates reference, and makes the work more serviceable to the practitioner. As now enlarged it is one of the most complete and comprehensive works of its class, and will meet the wants of the profession in this department most admirably. The authors have devoted considerable attention to the treatment of disease, have introduced the opinions of other authors, and given the results and investigations upon which their opinions are based, or the reasons which have induced to change their views and modes of treatment. Mercury in the treatment of cholera infantum and diarrhœa is nearly discarded. Of its use in pleurisy they quote from Horace Miller: "Formerly I gave mercury to all cases of primary pleurisy, but this practice I have discontinued, except in the form of an aperient. Instead of it, salines, such as acetate of ammonia, nitrate of potash or soda, the citrate of potash, and nitrous ether, are given." They then say: "The experience we have had since we last wrote has not at all increased our faith in this remedy. We believe that as time goes on, and knowledge grows, there is good reason to think that the good effects formerly ascribed to calomel in such a variety of diseases, were due to the medicine given with it, and particularly the opium, (without which it was not often used,) the ipecacuanha, the salines, and even the antimonials." Of blisters in pleurisy, they also speak in this wise: "Blisters are very generally applied in the acute form, to relieve pain and dyspnœa, and, in chronic, to hasten the absorption of the effused liquid. We did not apply them in the cases under our charge, having succeeded very well without." In this quiet and unpretending manner they try to educate physicians to discontinue the use of objectionable and unnecessary, not to say, injurious medication. But we suppose that several new editions of this and similar works will appear before great impression will be made upon former practices. It requires great force of argument to overcome established habits. We shall probably continue to give calomel to increase the biliary secretion for a hundred years after it is proved to have no such action, and to diminish, rather than promote it. We shall continue to apply blisters, and give antimony, and by various other means make sick people much sicker, until, as our author says: "Knowledge is greatly increased." This work seems to be very well up with present knowledge, old errors are carefully exposed, but old barbarisms are not attacked in some instances, with so efficient words as could be desired, and the former routine of treatment is repeated as though it were essential to the completion of the subject. We firmly believe, that at no distant day, the real value of therapeutic measures will be understood and independently expressed.

---

*Bennett's Practice of Medicine. Fifth American from the Fourth London Edition.* William Wood & Co., 61 Walker street, New York.

Not many months since we had occasion to speak of the work, and then gave as complete an idea of its character and scope as space would permit. Looking over the present edition we find addresses and improvements, better expressed by the author in his preface than otherwise. This work is in some respects, at least, more attractive than almost any other, both to the student of medicine and the general practitioner. It is better illustrated; takes in a wider range of subjects, and embraces the most recent investigations. A better idea of it will be gained by the following quotation from the author:

"In Section II. I have introduced an account of the molecular and cell theories of organization, and re-written descriptions of the general laws of nutrition and of enervation in health and disease, of inflammation, and of tuberculosis. In a note appended to the general treatment of morbid growths, I have inserted a letter from M. Velpeau, in which that distinguished surgeon has, from numerous cases in his practice, demonstrated the correctness of the opinion I long ago formed, on pathological grounds, viz., that true cancer may be permanently extirpated with the knife. The facts he has recorded ought to put an end to further discussion on the subject.

In Section III. I have introduced new considerations on the subject of General Therapeutics, and have referred, under distinct heads, to the natural progress of disease; the knowledge derived from an improved diagnosis and pathology; the fallacy of the change of type theory; an inquiry into our present means of treatment; and the proposition that physiology and pathology constitute the true foundation for medical practice. Regarding these subjects, which constitute important principles of our science, I shall be satisfied if their perusal should induce my readers to reflect on the uncertainty of our art, and stimulate some of them to renew investigations as to the exact value of remedies in the treatment of disease

In Sections IV., V. and VI., several new subjects, and many valuable cases, have been introduced, which it is hoped will render the account given of the diseases of the nervous, digestive and circulatory systems, more useful to the medical practitioner.

In Section VII. I have tabulated every case of acute Pneumonia treated by me in the Royal Infirmary of Edinburg since the year 1848, in order to satisfy my medical brethren that the restorative (not stimulating) treatment of the disease is in every way well worthy of their confidence. The facts shown by that table also will, I trust, serve to correct some prevailing errors, and establish a few new truths.

In Sections VIII., IX. and X. are many additional illustrations and new cases; some of the latter, illustrative of albuminuria, with increased secretion, from waxy degeneration of the kidney, are deserving attention. I trust to be excused for having still further defended my claim to the discovery of Leucocythemia. The subject of Diabetes has been extended by cases taken with great care, and a laborious trial of sugar as a remedy in that decease recorded. Certain views concerning the diagnosis and etiology of Typhus and Typhoid Fevers have been re-investigated. A very careful trial of the wet sheet in Scarlatina is detailed, and a singular new fact in the history of mercurial poisoning illustrated.

These, and numerous other additions, which it is calculated have increased the work to the extent of three hundred pages, I have, by curtailments, conpensation, the employment of closer type, and a slight enlargement of the page, been enable to effect without adding to the bulk of the volume."

---

*Glaucoma! A paper read before the Medical Society of the State of New York.* By HENRY D. NOYES, M. D.

This is a most excellent article upon Glaucoma and is now placed before many of our readers in the volumes of the Transactions of the New York State Medical Society. We bespeak for it a careful perusal.

---

*Transactions of the Nineteenth Annual Meeting of the Illinois State Medical Society.*

The volume of transactions of this Society is exceedingly creditable to the profession of the State. The reports of greatest interest are on Obsteterics, by H. B. Buck, M. D.; on Placenta Previa, by B. H. Cheney, M. D.; on Drugs and Medicines, by N. E. Davis, M. D.; Report on Insanity, by Committee; on Staphyloraphy, by Moses Gunn, M. D.; on Practical Medicine, by Committee; Supplemental Report on Epidemics, by D. L. Jewett, M. D.; on Extraction of Cataract, by J. S. Hildreth, M. D.; on Lenient Medication, by E. Ingals, M. D.; on Aphasia, by Henry Hurd, M. D.; Statistical Diseases of the Eye, by E. L. Holmes, M. D.; and report of committee on Necrology.

All these reports are well prepared and many are of very great value, on account of the original observation, and great care which has been bestowed upon them. We hope this society may long continue as active and prosperous as its learned volume of transactions now show it to be.

